# Foreign

**Published:** 1841-10-30

**Authors:** 


### FOREIGN.

*On the Curative Influence of Galvanism in some of the Organic Diseases of the Eye.* By Dr. Lerche, of St. Petersburg.—[The following is the report made by Dr. Lerche, one of the principal physicians of St. Petersburg, on the application to opacities of different parts of the eye of the galvanic action which Dr. Crusell, a physician of Finland, was said to have employed with great success for the removal of other kinds of thickening, &c., consequent on inflammation.]

To Dr. Crusell of Finland is due the honour of having employed galvanism in a new and peculiar manner for the cure of organic diseases. Having been attracted by the treatise on the subject which he had sent to the Imperial Academy of Sciences, I had proposed to repeat the galvanic experiments instituted by him on the diseases of the lens in animals. The necessary apparatus was not yet completed when Dr. Crusell himself arrived at St. Petersburg; we therefore determined to experiment in his presence and with his assistance. For the subject of the first experiment we chose a complete leucoma of the cornea, which was deemed incurable, and in which, therefore, nothing could be lost, and perhaps something might be gained. The patient, a boatman sixty-eight years old, was in our hospital for chronic inflammation of the other eye. Our apparatus, according to the directions of Dr. Crusell, consisted of a simple galvanic circle of one zinc and one copper plate, both immersed in dilute sulphuric acid. The wire proceeding from the copper plate, which we named the copper-pole, was placed in contact with the leucoma; that coming from the zinc-plate, the zinc-pole, was made to touch the patient's tongue, and the galvanic current was maintained for two minutes. The patient did not suffer in the least degree, neither did any unpleasant symptoms follow the operation; on the contrary, the chalky dulness at the margin of the cornea appeared somewhat thinner and clearer. The operation was repeated after three days, and we saw again a partial alteration in the state of the leucoma; and the patient, on his part, asserted that he had increased perception of light.

The idea was now not remote to apply galvanism for the cure of opacities of the internal tissues of the eye. The results obtained by Dr. Crusell, in his experiments on the eyes of animals, strengthened our expectation of doing good, and we had opportunities enough of repeating them on eyes which had been regarded as almost or quite incurable. For safety's sake, however, we determined first to see the effects of the operation on some animal, and a young pig was chosen for the purpose. A fine cataract-needle fixed on the zinc-pole was passed through the cornea into the crystalline lens of the right eye, and the wire from the copper-pole was stuck into the ear. After thus remaining for four minutes the pupil began to grow turbid, and the operation was discontinued on the right but repeated on the left eye. Some days after, we found completely-formed cataracts in both eyes, and the pig was quite blind.

Now, according to the theory, the artificially produced dulness of the lens ought to be removed by a reversed galvanic action, and before we could feel justified in undertaking similar experiments in human eyes it was necessary to obtain the proof that such would be the case. After ten days, therefore, we proceeded to this second operation. In three minutes, (the galvanic current being maintained through the wires which were now so placed that the copper-pole was connected with the eye and the zinc with the ear,) there appeared bubbles of gas in the pupil, and the process of solution seemed to be going on; the experiment was therefore at once brought to a close. The pupil appeared smoky and less dull. In four days it had almost regained its natural clearness; and the sight, so far as we could judge from the animal's action, was restored. In the cornea there was an opaque spot around the aperture made by the needle. This result at once determined us to make an experiment of a similar kind on the human eye.

We chose an old copper-smith from Finland, who had a long time previously been successfully operated on for cataract in the left eye. The right eye had presented a firm yellowish-brown capsulo-lenticular cataract, which had adhesions to the iris. It had been once depressed but it rose again; and in another attempt to break it up, nothing more could be done on account of its hardness, than the making of one vertical cut through its substance. This, however, rapidly closed, and for two months afterwards a variety of means were employed for the solution of the cataract without the least benefit. It was now very large, and lay close behind the widely dilated, somewhat irregular, and immovable pupil. The patient had a perception of light through it.

The galvanic operation was commenced in the presence of Dr. Crusell, in the forenoon of the 11th of November. It was very surprising to us to see, when the fine cataract needle fixed on the copper-pole was inserted into the lens, and the zinc-pole laid on the patient's tongue, how, in less than a minute, the cataract swelled up, increased in size, seemed to press against the cornea, and then suddenly burst into three peices, of which one was carried upwards and inwards, and another towards the temple, and the third projected downwards through the pupil into the anterior chamber. The triangular fissure left between them appeared clear and black. We now thought it best, as this was the first experiment of the kind on a living man, to stay the galvanic action. The patient could already see sufficiently to discern, with his right eye alone, both a finger held before it, and the face of a person standing in front of him. The operation lasted a minute, and neither produced pain or was followed by inflammation or any other bad symptoms.

Here the history ceases; the authors thought they had better leave the case with a limited amount of amendment, than run the risk of marring the advantage they had gained, and of throwing unnecessary discredit on the operation by going further. The facts, however, seemed to merit record as proofs that the plan is without danger, and offers some prospect of effecting a permanent cure of the disease.—*Brit. and For. Med. Rev. from Medicinische Zeitung*. Juni 16, 1841.

In these days, so rife in ill-digested schemes for worse than useless operations, it is almost dangerous to exercise the proper duties of a medical journalist by calling the attention of the whole profession to striking novelties, lest our notices should fall into the hands of those whose love of notoriety and its immediate monetary results renders them oblivious of the honourable laws of professional trust. There are those who will be ready enough to seize upon the idea growing out of the experiments of Dr. Crusell, and acted upon by Dr. Lerche and himself, without the knowledge and discretion necessary for the regulation of such an agent as galvanism, when employed upon the delicate tissues of the interior of the eye. We should not be surprised to hear of the *explosion* of many crystalline lenses, within a few months, among our restless and enterprising countrymen. The whole subject of galvanism and electricity is beautifully obscure, and admirably adapted to the purposes of intra-professional empiricism, as well as that which flourishes so vigorously among those who make no claim to fellowship with us. If, then, as well may happen, our daily papers should hereafter furnish us a daily dish of operations in which the lens had been *splendidly* "burst," and the pieces "projected" to a distance by the agency of wonder-working galvanic plates and wires, it is desirable that some kind friend of humanity should be upon the watch, to let us know the ratio of ruined eyes resulting within the three weeks following the *display*. The official bulletins of such transactions usually relate no more of the history of such cases than is witnessed at the moment. A little narrative of ultimate results would be the death of many an ephemeral glory in surgery, that now lives long enough to cause much human suffering, and undermine the public faith in medical ability.

Our object in these remarks is not *to condemn*, but only *to caution*, for we all are well aware that electricity and galvanism are important remedial agents, and regard the experiments of Drs. Crusell and Lerche as deeply interesting. It is to be regretted that the extract contains no more particular account of the apparatus employed, and especially that no statement of the size of the plates of zinc and copper is given. This information we will endeavour to obtain for our readers as soon as practicable. The great fault in most of the applications of imponderable agents to the treatment of disease has been the use of undue power. We well remember to have succeeded, almost immediately, in curing a local-paralysis of the arm in a case in which the disease had been extended and exacerbated by shocks from an eight-ounce Leyden phial. Our only remedy was the application of shocks from a phial of one ounce! Even in galvanic experiments upon the recent dead, the purpose of the operator has been frequently thwarted, in great degree, by the unnecessary energy of the pile, which has crushed, destroyed, or thrown into an instantaneous collapse, the nervous susceptibility which it was designed to stimulate.

The effect of electricity and galvanism upon the process of absorption has seldom been the subject of well-ordered experiment. That the former has a powerful influence in promoting secretion is well known. Sparks drawn from the body of a patient seated on an insulated chair may be made very readily to double the discharge of a seaton or issue; and, many years ago, we freely employed this method with advantage in the practice of the Pennsylvania Hospital. If the process be repeated by drawing the sparks daily from the same spot for a week, there usually follows a free crop of pustules, resembling those produced by the plaster of tartar emetic. The stream of electricity flowing from a point upon the cornea was formerly recommended in leucoma by the advocates of this class of remedies, and Singer and others claim to have effected cures in this manner. In several faithful trials, we have failed in the attempt to make any impression on the opacity. But it would appear that, in the experiments of the Fin and Russian observers, it was the negative and not the positive pole that incited the absorbent process, while, in the electric trials, as far as our recollection of authorities extends at present, the positive fluid was employed in all the cases.

The plan proposed would be perfectly safe, as well as easy of application on opacities unattended with inflammation, provided the zinc and copper plates were small; but in the action of acupuncture on the lens, it would appear, from the "bubbles of gas in the pupil," that actual chemical decomposition was effected. It would not require the sudden distention of any great quantity of gas in the eye to endanger paralysis of the retina by compression; and, independently of the probability of inflammation, this circumstance might require some caution in the employment of plates of much power. To say the least of this seemingly formidable operation, common prudence obviously demands a full trial of the apparatus upon opacities, so as to establish some clearer views of its modus operandi, before venturing upon the far more dangerous puncture of the lens performed by Dr. Lerche. Such experiments do not require hasty action, and the surgeon may well wait a few months for further information, before running the unnecessary risk of tampering unwisely with parts so delicate as those about a cataract.

---

The careful observer of the current of scientific discovery, may now perceive the dawning of another era in the progress of medical theory. The tendency of chemical investigation is towards the discovery of such affinities as may account for many of the hitherto occult steps in the processes of absorption, deposition and other vital phenomena upon principles purely chemical; and while analysis encroaches upon the dark domain of the *vital principle*—that high sounding apology for ignorance—on the one hand, the microscopic observers, the vegetable physiologists, and the special naturalists, are beginning to attack our established notions of transcendental vitalism in other directions. Time was when it was held that living tissues could not tolerate the contact of living bodies of another species unless inclosed in cysts or contained in cavities, but now we know that, in diseased conditions of the vegetable and animal frame, the germs of other individuals may rest inactive, or may grow and flourish for long periods of time without destroying the individual on which the parasites remain engrafted. The various forms of dry rot in woody fibre are believed to be cryptogamous plants, and some of them are seen in full activity—diffused, and not incysted—within the substance of living but sickly trees. Some years ago, the naturalists of the north were thrown into general agitation by the announcement of the discovery of an insect at the south whose body in dying (oh! shade of Narcissus!) converted itself into a flower! Specimens were forwarded to the Academy of Natural Sciences. They proved to be the undecayed bodies of mole crickets or grillo-talpæ,—whose vanity must have been indeed preposterous if it could induce them to rival, with their ungainly figures, the beautiful Grecian boy! From each of these sprung forth a tall and somewhat complex cryptogamous plant; bearing no inconsiderable resemblance to a flower. It was the general opinion that the germs had been taken by the animals with their food; that they had retained their vitality and had germinated immediately after the death of the insect. Rumor asserts that their growth preceded the fatal moment, and that these gnome-like denizens of the soil were compelled to visit the upper air, bearing about upon their shoulders the foreign trophies of another kingdom until their juices were absorbed and they sunk beneath the weight of their undesirable decorations. In these days, when certain modern schools of philosophy lean too strongly towards materialism,—if, as the following extracts seem to show, our living bodies may be made the food of cryptogami, it is certainly time to review some of our physiological opinions and brush up our *mouldy ideas!*

*On the development of Cryptogamia in the tissues of living Vertebrata.* By MM. Rousseau and Serrurier.—In a memoir on the diseases of the organs of the voice by the above gentlemen, it is related that a male paroquet affected with laryngeal and pulmonary phthisis died in 1834, and on examination there was found in the abdomen between the intestines and vertebral column a species of false membrane, on which there was a greenish and pulverulent mouldiness, which adhered so slightly that on blowing it disappeared as the finest and lightest powder. This mouldiness has been several times observed in different parts of the body; most frequently in the pelvis, between the kidneys and the viscera, on the principal vessels of the heart, between the ribs and the lungs. Pigeons and fowls are most particularly affected, especially those inhabiting cold and moist places, and in rainy seasons. Nevertheless this phenomenon has occurred in animals of a different organization, in a hind (*cervus axis*) and in the earth tortoise, a native of the Indies (*tesdudo indica.*) A similar fact has recently been communicated to the academy by M. Deslongchamps.

At the sitting of the Royal Academy of Sciences, July 5, 1841, M. Dumas stated that Gruby had discovered that one of the varieties of porrigo is owing to the development of a vegetable beneath the epidermis. M. Breschet remarked that the *porrigo favosa* was the form studied by M. Gruby.

*Gazette Médicale. Juillet* 17, 1841.

M. Kettner has since written to the Academy to claim for Dr. Schönlein, successor to Hufeland at Berlin, the priority of indicating the existence of a vegetable in the crusts of *porrigo favosa*, a fact which is published by him in *Müller's Archiv.* M. Kettner states that Dr. Schönlein has established in many cases of *porrigo lupinosa* the vegetable nature of this affection, and therefore the discovery cannot be attributed to M. Gruby.

*Gazette Médicale. Juillet* 24, 1841.

M. Gruby has made very minute observations on the physical and chemical properties of this vegetable, and has succeeded in innoculating it on cryptogamia, thus transmitting disease from man to vegetables, but only succeeded once in seventy-six trials. He failed to communicate the disease by inserting the vegetable into the arm. This subject is so interesting and important in connexion with the subject of animate contagion, that we shall wait with anxiety until the report of the committee is published.—*Ed. Rev.*

---

*On Syphilis in Pregnant and recently-delivered Females.* By M. Huguier.—M. Huguier has made a long series of observations on this subject, and has arrived at the following conclusions:

1. The sole primitive phenomena of syphilis is sometimes a pustular eruption at the vulva. This is often the commencement of chancre. 2. Purgatives are not proper for pregnant females tainted by syphilis. 3. Abortion in syphilitic females is rather in consequence of the exhibition of mercury than of the disease itself. 4. The venereal disease has not the grievous influence on pregnant females which is attributed to it. In twenty-seven females in this condition, only three are dead, and probably their death resulted from lesions unconnected with syphilis. 5. The mercurial treatment does not always preserve the infant nor protect from relapses, and often produces serious results. 6. The sublimate is the most dangerous among the mercurials, and mercurial frictions are preferable to it. 7. Syphilis is very often hereditary; and it ordinarily declares itself from three to thirty days after birth. This last remark is important, for if the child be examined on the first or second week, it is declared healthy and sent out to nurse, probably with the effect of infecting the female.—*Ibid.*, *from Archives Gen. de Med.*

